# The reliability of bilateral cerebral laterality for word generation: Who is left in the middle?

**DOI:** 10.1162/IMAG.a.102

**Published:** 2025-08-07

**Authors:** Robin Gerrits, Guy Vingerhoets

**Affiliations:** Research Group Cognition and Plasticity, Max Planck Institute for Human Cognitive and Brain Sciences, Leipzig, Leipzig, Germany; Department of Experimental Clinical and Health Psychology, Ghent University, Ghent, Belgium; Ghent Institute for Metabolic and Functional Imaging (GIfMI), Ghent University, Ghent, Belgium

**Keywords:** functional transcranial Doppler sonography, hemispheric specialization, laterality, visual half field task, language dominance, test-retest reliability

## Abstract

Although word production is considered a strongly left hemispheric lateralized function, its cerebral asymmetry varies among individuals. The most popular way of determining hemisphere dominance is to calculate a laterality index (LI) by comparing brain activity between the two hemispheres. Large LIs can readily be classified as left or right dominant, but there is no consensus on how to treat bilateral LIs indicating (near) symmetrical activity. The problem with interpreting very small LIs is perpetuated by a lack of reliability, stemming from the challenge of systematically investigating these uncommon cases (usually ≤ 10% of a sample). To address this gap, we performed two studies that investigated the reliability and across-methods generalizability of bilateral LIs obtained from functional transcranial Doppler sonography (fTCDS)—an ultrasound-based approach that measures cerebral blood flow velocity. In Study 1, we compared reproducibility of bilateral LIs (n=35) and clearly lateralized LIs (n=32) during a letter verbal fluency fTCDS task across two sessions. While left-lateralized classifications were highly replicable (97% reproduced), poorer reproducibility was observed for bilateral classifications (51% reproduced). In fact, dichotomous left-right categorization yielded more reliable outcomes than assigning participants with bilateral LIs to a separate category (80% reproduced). Study 2 assessed whether small fTCDS asymmetry extended to other instruments for determining cerebral laterality (visual half-field method and fMRI). Participants consistently classified as bilateral by fTCDS (n=18) also exhibited reduced group-level asymmetry in these other methods. Based on these results, we suggest that LIs reflect a combination of idiosyncratic cerebral asymmetry, state-dependent fluctuations, and measurement noise. Our findings also indicate that a subset of the population has a neural system for word production that is inherently weakly lateralized, although true hemispheric equivalence is likely extremely rare. Finally, we offer recommendations for classifying asymmetry in clinical and research contexts.

## 1 Introduction

The neural system supporting language generation is best described as bihemispheric yet lateralized (Price, 2012; Riès et al., 2016). Although usually both halves of the brain are engaged when we produce words and sentences, it is the integrity of the left hemisphere that is crucial for typical language performance in the vast majority of people. Strokes and other injuries to the left cerebral hemisphere often cause chronic language deficits (aphasia), whereas right-sided damage seldom results in aphasia (Carey & Johnstone, 2014; Coppens et al., 2002). Similarly, when brain activity is unilaterally suppressed during Wada testing as part of pre-operative assessments of language dominance in clinical populations, speech arrest and other transient language impairments are typically apparent only after sedating the left hemisphere (Rasmussen & Milner, 1977). The invasive nature of the Wada test restricts its use to patients with specific neuropathologies that would benefit from this procedure, such as epilepsy, and has nowadays largely been supplanted by less risky and less burdensome alternatives, such as neuroimaging (e.g., fMRI) or neuromodulation (e.g., TMS) (Bruzsa et al., 2023; Qadri et al., 2021). Studies using fMRI, TMS, and other non-invasive methods have consistently demonstrated a robust left hemispheric dominance for language production in the intact brain, with approximately 90% to 95% of individuals exhibiting this pattern (Knecht et al., 2000; Mazoyer et al., 2014; Szaflarski et al., 2006; Tussis et al., 2016).

The most common method for determining hemispheric (language) dominance at the individual level, for research and clinical purposes alike, is to record task-evoked brain activation and calculate a laterality index (LI) that expresses the difference in neural activity between the hemispheres. Performing expressive language tasks typically results in pronounced asymmetries in brain activity for most individuals, leading to relatively large LIs, which makes it straightforward to classify these individuals as left or right hemispheric dominant. However, classification becomes progressively more difficult as the LI approaches zero, indicating increasingly symmetrical activation, since there is no consensus approach for dealing with ‘small’ LIs (Vingerhoets et al., 2023). Some authors simply classify a participant as left dominant as soon as their left hemisphere is slightly more active than the right, and vice versa, essentially basing their decision on the sign of the LI while ignoring its absolute value (e.g., Johnstone et al., 2020). Others regard LIs below a certain threshold as unreliable measurements that do not allow drawing definitive conclusions about an individual’s hemispheric dominance. This perspective is typically recommended in clinical decision-making during presurgical assessment of language dominance, where bilateral fMRI findings are often seen as inconclusive and indicate that a more invasive procedure, such as Wada testing, is required to ascertain the patient’s language laterality (Qadri et al., 2021). Still others believe that individuals with small LIs form a separate group with a categorically different form of language organization in which neither hemisphere is dominant (e.g., Mazoyer et al., 2014). The idea of such a “bilateral” language system has its precedence in the Wada test, during which the occasional patient was observed to lack hemispheric language dominance, either because they demonstrated a similar degree of language impairment or suffered no apparent language symptoms, irrespective of which hemisphere was sedated (Bernal & Ardila, 2014).

Even though participants with small LIs typically constitute only 5% to 15% of a given sample (Knecht, 2000; Mazoyer et al., 2014; Szaflarski et al., 2002), their classification poses a major methodological issue and making the ‘wrong’ choice could have dire ramifications. If small LIs indicate true (near-) symmetrical language organization, adopting a dichotomous left-right classification scheme would erroneously group individuals who lack hemispheric specialization with those who are lateralized. This misclassification could diminish the statistical power to detect effects driven by functional lateralization. If small LIs are reliable and valid measurements, the cautious approach of excluding participants with small LIs would actually lead to the unnecessary loss of valuable data and neglect genuine variations in cerebral laterality. On the other hand, if small LIs generally do not reflect actual symmetrical language organization, the addition of a bilateral category would be unfounded and might lead to false conclusions about hemispheric language organization. Moreover, the heterogeneity in strategies for handling small LIs and the different cut-offs that are chosen to decide bilaterality in trichotomous classification schemes make it challenging to compare and integrate the rates of left, right and bilateral language dominance across different studies.

One issue impeding consensus on how to classify small LIs is the paucity of reliability studies from which recommendations could be drawn. Strong replicability of the direction, but not the degree, of small asymmetries in brain activity would be an argument in favor of a dichotomous classification. In case neither direction nor degree is reproducible, excluding individuals with small LIs seems warranted. Finally, treating participants with small LIs as a separate bilateral group would only be defensible if their reduced asymmetry replicates across different test sessions. While plenty test-retest studies have found that task-derived language LIs are moderately to highly reproducible, these studies recruited right-handers exclusively or gathered unselected participant cohorts. As a consequence of the uncommonness of ‘weak’ language asymmetry, particularly amongst dextrals, their samples mainly consisted of individuals with strong left lateralized LIs (Bishop et al., 2009; Jansen et al., 2006; Nettekoven et al., 2018; Rutten et al., 2002; Stroobant & Vingerhoets, 2001; Wilson et al., 2017). As such, these studies are not informative about the reliability of weakly lateralized LIs specifically. Only one study explicitly mentioned poor inter-session reproducibility of bilaterality, but included only 10 participants in total and did not report how many were considered bilateral (Jansen et al., 2006). A re-analysis of split-half reliability data also suggests that bilaterality might not replicate well across two fMRI runs (Johnstone et al., 2021). The same caveat applies here, however, as only seven participants had an LI that could be deemed bilateral during the first run (LI ≤ 20%). Finally, one other hemodynamic imaging study reports good replicability for a subgroup of nine participants classified as “atypically lateralized” (Hodgson & Hudson, 2017). However, this subgroup contains a mixture of participants classified as bilateral and strongly right-lateralized, making it impossible to draw conclusions specific to small LIs.

At present, there are insufficient data available to judge the reliability of small LIs specifically. A major rationale for the research reported in this article is to fill this gap. Achieving this requires overcoming the practical hurdle of gathering a sizeable group of participants with small LIs, which necessitates recruiting a large cohort of participants given the low prevalence of individuals with small LIs. Using the technique regarding as the common standard for this purpose, that is, fMRI, would be time-consuming and prohibitively expensive. Therefore, the current study opted for a more cost-effective and time-efficient strategy that relied on a closely related, but cheaper, hemodynamic method called functional transcranial Doppler sonography (fTCDS) (Hartje et al., 1994; Rihs et al., 1995). With fTCDS, task-evoked dynamics in cerebral blood flow velocity (CBFV) are measured within the middle cerebral artery (MCA), which are inferred from frequency changes in emitted and reflected ultrasound signals due to the Doppler shift (Deppe et al., 2004). An increase in CBFV indicates a rise in neural activity similar to the BOLD in fMRI (Stroobant & Vingerhoets, 2001). Doppler-based LIs can be obtained by subtracting changes in CBFV between the left and right MCA. Although fTCDS, unlike fMRI, cannot precisely localize brain activity, it is inexpensive, fast, can yield measurements that are stable across repeated sessions, and agrees well with other methods for determining hemisphere language dominance, such as fMRI and the Wada test (Deppe et al., 2000; Knecht et al., 1998; Stroobant & Vingerhoets, 2001; Vingerhoets & Stroobant, 2002). By using fTCDS as a screening procedure to find individuals with small LIs, the current research aims to (1) examine the test-retest reproducibility of small LIs as well as the inclusion of a “bilateral” categorize to classify hemispheric dominance (Study 1), and (2) to assess whether consistently reduced asymmetries identified by Doppler generalize to other methods for establishing language laterality (Study 2). In Study 1, participants performed a letter verbal fluency task—one of the most frequently used paradigms to determine hemisphere language dominance—at two different timepoints at least 1 week apart while their cerebral blood flow asymmetry was recorded using fTCDS. In Study 2, cerebral language asymmetry was examined in participants from Study 1 who consistently demonstrated bilateral or clear left lateralized dominance in both fTCDS sessions using two other common methods: the visual half-field method and fMRI.

## 2 Study 1: The Test-Retest Reliability of Bilateral “Dominance” using FTCDS

### 2.1 Methods

#### 2.1.1 General procedure, recruitment strategy, and inclusion criteria

Study 1 was preregistered on Open Science Framework (OSF, https://osf.io/2mb9t). Individuals with small LIs (“bilaterals”) and clearly left lateralized controls were recruited through their participation in three other, as of yet unpublished, fTCDS studies whose protocol included the same letter verbal fluency paradigm. Briefly, the first study aimed to chart variability in hemispheric organization of speech production, manual praxis, and spatial attention (described in more detail here: https://osf.io/hs6kq/); the second examined performance-laterality associations in language production; and the third sought to validate Doppler paradigms for mathematical cognition. Each individual classified as bilateral for verbal fluency was asked to participate in a follow-up session scheduled 1 to 8 weeks later, during which they repeated the Doppler verbal fluency task. Participants in those three studies were reached via the University’s Research Participation System, advertisement on social media, and flyering. The eligibility criteria to participate in the current study were (1) age between 18–40 years, (2) native Dutch speaking, (3) normal or corrected-to-normal vision, (4) absence of neurological disease, major psychiatric disorder, or diagnosed neurodevelopmental disorder. The letter verbal fluency task was completely identical in all three studies. Participants were invited to the follow-up fTCDS session if (1) they provided informed consent to be approached for participation in future studies, (2) they were classified as bilateral based on a silent letter verbal fluency fTCDS task (as defined below), or (3) they were classified as left dominant during this task and could be matched to an already re-tested participant in the bilateral subgroup in terms of age (≤3 years difference), handedness, and biological sex. Written informed consent was obtained from each participant in every test session. The Ethics Committee of the Faculty of Psychology and Educational Sciences of Ghent University gave ethical clearance for this study before the data collection began (approval number: SEP 2021-213).

#### 2.1.2 fTCDS procedure and data processing

Fluctuations in cerebral blood flow velocity (CBFV) during covert word generation were recorded using a Multi-Dop T transcranial Doppler sonography device (*DWL*) running QL software version 3.6.7. A DiaMon headset (*DWL*) with two 2Mhz ultrasound monitoring probes was mounted on the participant’s head. These ultrasound probes were manually placed on the left and right transtemporal window in order to reach the middle cerebral artery (MCA) at a conventional depth of insonation (40–60 mm). Given that variability in skull bone density and thickness impacts the quality of the received Doppler signal (Wijnhoud et al., 2008), the intensity of the emitted ultrasound (i.e., “gain”) was individually adjusted to ensure optimal transmission through the acoustical window, minimizing the signal-to-noise ratio based on visual inspection of the signal. When no apparent signal was found at the default amplification level (gain = 36), the parameter was systematically increased up to a maximum of gain = 56. If no signal could be identified on either side after 30 min of searching at this maximum gain, the experiment was aborted, and the participant excluded. In case MCA-sampling necessitated insonation depths exceeding 60 mm, the decision to consider the signal originating from the MCA was based on the shape of the waveform and mean blood flow velocity ranges. Blood flow velocity in the MCA is, on average, higher than in other vascular structures that can be insonated through the transtemporal window, such as the posterior cerebral artery and terminal intracarotid artery (Lupetin et al., 1995). In cases of uncertainty, the participant’s data were excluded, and the MCA recording was treated as missing.

After the MCA was located and the ultrasound probes were fixed, participants completed a word generation task in which they had to silently think of as many words as they could beginning with a letter shown in the middle of a laptop screen. We opted for a silent task, rather than an overt version, to minimize movement-related artifacts, prevent auditory self-feedback from confounding lateralized brain activation measurements, and ensure comparability between the fTCDS and fMRI paradigms (Gutierrez-Sigut et al., 2015). Word generation trials lasted 20 s and were preceded by a 3 s instruction screen (‘Clear your mind’) and 15 s rest trials indicated by a central fixation cross. Participants were asked to think of nothing in particular during the rest period to allow the CBFV to return to baseline after the word generation phase. Sixteen task trials were presented, each involving a different letter (S, B, K, V, P, A, O, T, G, R, M, D, H, L, W and Z). The experiment started with a practice trial to familiarize the participant with the task (letter N). The task was run on a Dell laptop (Windows 10) using PsychoPy version 2021.2.3 (Peirce et al., 2019).

The fTCDS data were processed in R using a script adapted from Woodhead et al. (2021). First, the recorded CBFV was down-sampled from 100 Hz to 25 Hz and subsequently segmented into 38 s epochs, spanning 10 s before stimulus onset until the end of word generation trial. Next, spikes and dropouts were detected (i.e., CBFV values outside of the 0.0001–0.9999 quantiles). If only one such outlier was present within an epoch, that value was replaced by the mean for that epoch. If multiple artefacts were identified, the epoch was excluded. A visual inspection to identify and reject additional artefacts was then performed on the epoched data. To ensure the CBFV was independent of the angle of insonation and the MCA diameter, normalization was performed by dividing each CBFV value for the left and right hemisphere separately by its mean and multiplying this by 100. Confounds due to heart beat were removed by heart cycle integration, and epochs were next baseline corrected using the 8 s of rest preceding the instruction screen as a baseline. Epochs with normalized CBFV values lower than 60% or higher than 140% were removed.

Based on all retained epochs, a laterality index (LI) was calculated expressing the average difference between the CBFV within the left and right MCA during a 17 s period-of-interest spanning 3 s after the start of word generation phases until the end of the task period. In addition, two split-half LIs were obtained using only the odd trials or only the even trials. Finally, a participant-specific standard error (SE) of the LI was calculated, capturing the epoch-to-epoch variability in laterality. This standard error served to construct a confidence interval used to classify participants in a data-driven way. If the 95% confidence interval around the LI included zero, the participant was classified as bilateral language dominant (BLD). Otherwise, they were classified as left language dominant (LLD) if the LI was negative or right language dominance if the LI was positive.

### 2.2 Statistical analysis

The preregistered goal of Study 1 was to assess the test-retest reliability of (1) small LIs from fTCDS and (2) including a “bilateral” (BLD) category for classifying hemispheric language dominance. To examine the reliability of the LIs, intra-class correlations (ICC) were calculated between the LIs from test sessions 1 and 2 for the full sample and separately for participants initially classified as BLD and LLD. A two-way mixed-effects model with single measurement and absolute agreement was used. Complementary to this approach, the reliability of the categorization was assessed by calculating the percentage agreement of classifications between sessions for the full sample and the initial LLD and BLD subgroups. Cohen’s Kappa was used for whole-group analysis to measure inter-session reliability, while uncorrected percentage agreements were calculated for the BLD and LLD subgroups. In addition, Bland-Altman plots were used to illustrate measurement variability and identify potential systematic biases in the LI measurements across the two fTCDS sessions (Bland & Altman, 1986). These plots visualize the differences between the two LIs as a function of their mean value, as well as the limits of agreement (±1.96 standard deviations from the mean difference), which indicate the range within which 95% of differences are expected to fall.

Another approach to classify LIs, besides the data-driven CI-based method, uses a fixed threshold above which participants are classified as lateralized and below as bilateral. In another analysis, we therefore explored the impact of cut-off selection on the test-retest reliability of bilaterality by calculating the percentage agreement over a range of LI cut-offs (LI ≥ 0 to 1.5, with steps of 0.1).

### 2.3 Results

#### 2.3.1 Participant sample

A total of 376 participants were considered for the initial inclusion in the fTCDS screening part of the study, of whom 227 self-identified as left-handed. Doppler signals from the bilateral MCA could not be obtained in 13 participants (3.5%) who were excluded along with 24 participants who reported they were diagnosed with a developmental disorder. Verbal fluency fTCDS was recorded from the remaining 339 participants. After removal of epochs with poor data quality, four participants with an insufficient number of remaining trials were excluded from further analyses. The 335 retained participants (208 left-handers, 62%) were screened for bilaterality. Based on their fTCDS task LI, 42 participants were classified as BLD (12.5% of the full sample), 29 of whom were left-handed (69% of all BLD), 262 participants were categorized as LLD (78.2% of the full sample), and the remaining 44 were classified as right hemispheric dominant. The proportion of participants classified as BLD was 20.2% (42/208) in the left-handers and 10.2% (13/127) in the right-handers.

**Fig. 1.**
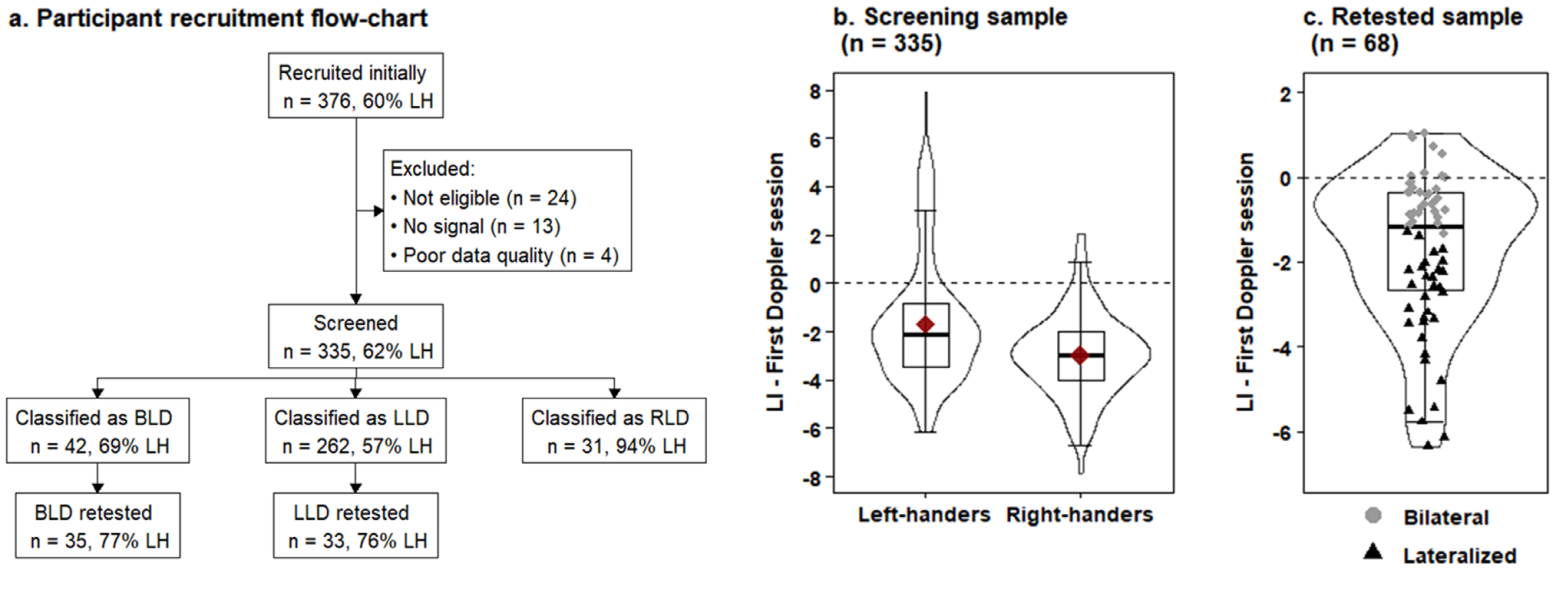
Participant recruitment flow and distribution of Doppler laterality indices in Study 1. (a) Outcome of participant recruitment in Study 1. LH = left-handers, BLD = bilateral language dominant, LLD = left language dominant. (b) Distribution of the laterality indices (LI) in the first Doppler session in the full sample screened for bilaterality, shown separately for left-handers and right-handers. (c) Distribution of the laterality indices (LI) in the first Doppler session in the subset of participants who were retested. The boxplots in panel b and c span the 25^th^ to 75^th^ percentile, with the median indicated by the thick black line. Negative LI values represent leftward asymmetry and positive values represent rightward asymmetry. The red diamond in panel (b) indicates the average.

The second Doppler session included 35 participants with an initial BLD classification and 33 participants initially classified as LLD. The average time interval between the two Doppler sessions was 13 days (standard deviation (sd) = 8 days) and this interval did not differ significantly between participants with an initial BLD classification (mean = 12 days, sd = 8 days) and an initial LLD classification (mean = 14 days, sd = 8 days), Welch two-sample t-test, t(65.86) = -0.860 days, p = 0.393. Sample demographics are shown in Table 1. The participant flow diagram is shown in Figure 1a together with the distribution of the verbal fluency LIs in the full sample (Fig. 1b) and in the subgroup of participants who joined the retest Doppler session (Fig. 1c).

**Table 1.** Demographics of participants in Study 1.

	Full sample	Initial BLD	Initial LLD
	*(n=335)*	*(n=35)*	*(n=32)*
Age, mean ± sd [years]	20.6 ± 3.64	20.42 ± 3.49	20.03 ± 2.65
Sex [% Female]	81.5%	88.6%	87.50%
Handedness [% Left]	62.2%	77.1%	75%

BLD = bilateral language dominant participants, LLD = left language dominant participants.

#### 2.3.2 Preregistered analyses

In a first analysis, the inter-session reliability of categorizing hemispheric dominance during verbal fluency based on fTCDS was assessed. For the full retest sample (n = 68), the corrected percentage agreement expressed by Cohen’s kappa was 0.477 (Z = 4.45, p < 0.001), indicating moderate agreement. Decomposing this result for the initial LLD and initial BLD groups separately revealed that only one participant initially classified as LLD (1/33, 3%) switched to the bilateral class based on the second test session and that the sign of the LI did not flip in any of them. By contrast, only about half of the participants with initial BLD demonstrated an LI deemed bilateral in the follow-up session (18/35, 51% percentage agreement). In the remaining participants with initial BLD, 16 were classified as left hemispheric dominant (46%) and one as right hemispheric dominant (3%) in the retest session. The sign of the LI and thus dominantly activated hemisphere changed in 28 out of 35 participants with initial BLD (80%).

**Fig. 2.**
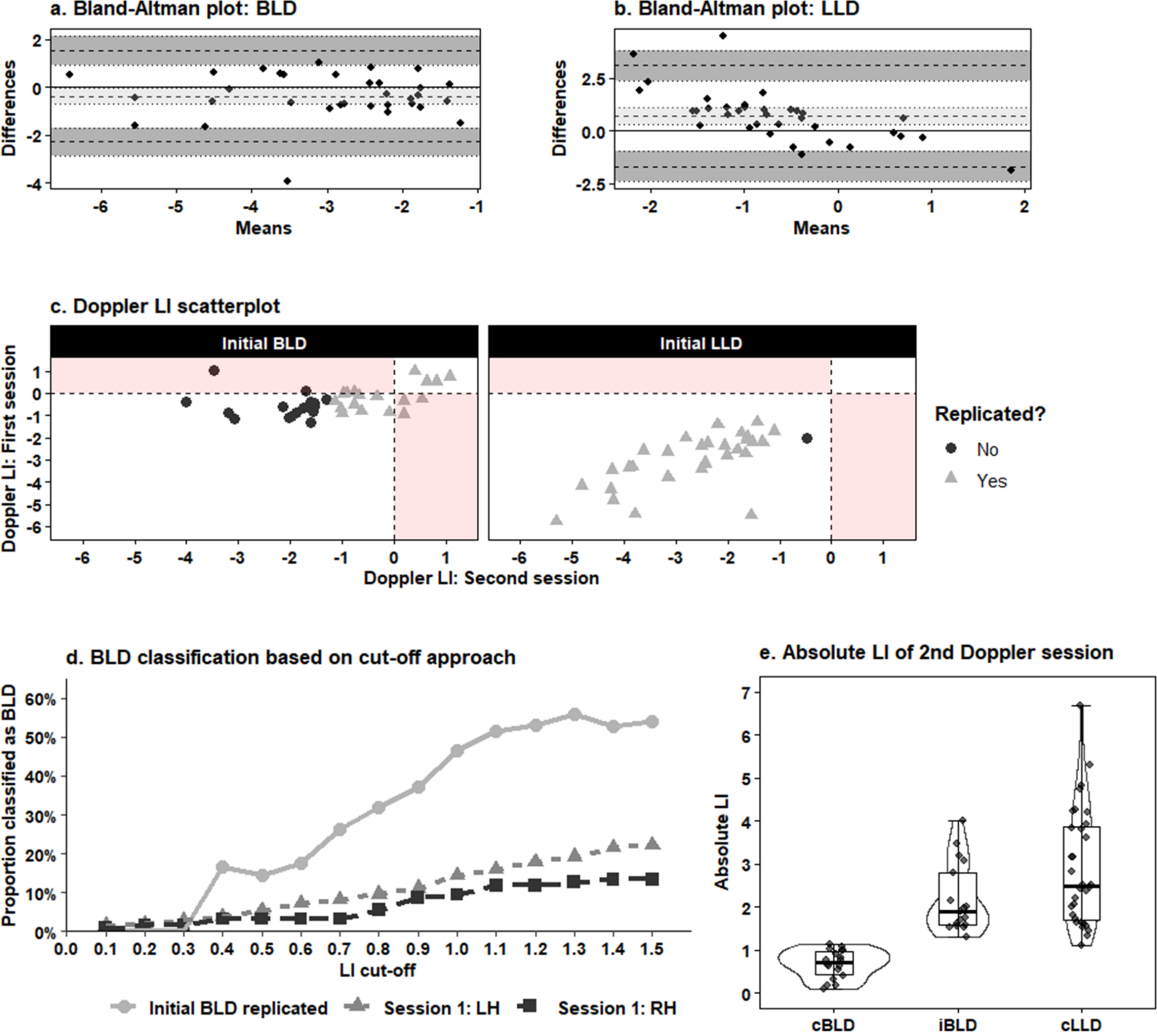
Test-retest reliability of laterality indices and classification across Doppler sessions. (a) and (b) Bland-Altman plots of the difference in fTCDS LIs between the first and second session in the bilateral language dominant (BLD) and left language dominant (LLD) participants respectively. The solid line indicates the mean difference. The dotted lines represent the 95% statistical limits of agreement. (c) Scatterplots of the fTCDS LIs from the first and second test session. Negative LI values indicate leftward asymmetry, positive values indicate rightward asymmetry. Shaded areas represent discrepancies in the sign of the LI (i.e. direction of laterality) between the two sessions. (d) Proportion of individuals classified as BLD when fTCDS LIs ≤ a fixed cut-off are categorized as bilateral. Circles represent the proportion of participants whose bilateral classification replicated across test sessions. The triangles and squares represent the proportion of bilateral classified left-handers (LH) and right-handers (RH) respectively in the full fTCDS sample. (e) Comparison of the absolute value of the LI between consistent bilateral language dominant (cBLD), inconsistent bilateral language dominant (iBLD) and consistent left language dominant (cLLD) participants. The boxplots span the 25^th^ to 75^th^ percentile, with the median indicated by the thick black line.

The ICC between the fTCDS LIs in the first and second test sessions was 0.746 for the whole sample, which was significantly higher than an absent correlation, F(67,68) = 6.91, p < 0.001, and indicates moderate to good test-retest reliability. In the initial LLD group, the ICC was 0.739, which also differed significantly from zero, F(32,26.9) = 7.3, p < 0.001. Participants initially classified as BLD demonstrated an ICC of 0.294, which, while significantly higher than zero, F(34,25.5) = 2.08, p = 0.029), indicates poor reliability. Bland-Altman plots and scatter plots of the LIs in sessions 1 and 2 are shown by Figure 2a-c. Bland-Altman plots and scatter plots of the LIs in sessions 1 and 2 are shown by Figure 2a-c. The Bland-Altman plot of the LLD group indicates there is no substantial bias between the LIs of the two test sessions. The between-session variability is acceptable since the differences tend to stay within the 95% limits of agreement, suggesting consistent test-retest reliability. In contrast, for the BLD group, a downward trend is observed, indicating a tendency for the second session to produce larger LIs compared to the first. The between-session variability is noticeably larger than in the LLD group, although it tends to remain within the 95% limits of agreement, This suggests that, while there is no extreme inconsistency between the two sessions, the reproducibility is less stable compared to the LLD group. The results of the alternative cut-off based approach for classifying participants as bilateral versus lateralized is shown by Figure 2d.

#### 2.3.3 Non-preregistered analyses

We performed a series of follow-up analyses to examine possible explanations for the group differences in test-retest reliability indicated by the main analysis. First, we assessed whether the reduced reliability may have reflected a general increase in cerebral laterality attributable to performing the task twice (e.g., training effects). Such an effect would not impact the classification of individuals initially classified as clearly left hemispheric dominant, but could push participants categorized as BLD during the first test session to become lateralized during the retest-session. To that end, a two-tailed, paired t-test was performed to compare the LIs of the LLD group between the first and second session. This test revealed a non-significant trend towards a lower LI in the first test session (mean LI = -3.23, sd = 1.41) compared to the second test session (mean = - 2.89, sd = 1.36), t(31) = 1.95, p = 0.06, indicating that cerebral asymmetry does not become more pronounced when repeating the verbal fluency task. Second, we examined whether (in)consistent BLD and LLD participants differed in terms of the settings of the Doppler recordings. Two parameters of the recording need to be adjusted individually each session to account for variations in vascular and cranial anatomy as well as unavoidable between-session differences in the exact probe positions: the insonation depth at which the CBFV is recorded and the ‘gain’ (i.e., amplitude) of the emitted sound. To rule out that the decreased test-retest reliability in the BLD group may have resulted from systematic group differences in these parameters, a series of t-tests were conducted to compare the mean depth and gain of the left and right probe between inconsistent BLD, consistent BLD and consistent LLD participants. None of these tests yielded a significant result, as further detailed in Supplementary Materials 1. Lastly, using a series of t-tests, the SE of the LI was compared between the same participant groups since a higher SE, which captures trial-to-trial variability in the lateralization of the CBFV, could indicate the measurement was more noisy. Once again, none of the comparisons reached statistical significance (see Supplementary Materials 2), indicating trial-to-trial variability of the Doppler recordings did not differ between consistent BLD, inconsistent BLD and consistent LLD participants.

For those initial BLD participants whose bilateral classification did not replicate, it might still be the case that they are less lateralized in the retest session compared to the consistent LLD participants. To examine this, a one-sided Mann-Whitney U test on the absolute LI of the second Doppler session showed that the absolute LI was significantly smaller in the inconsistent BLD group (median = 1.89) than in the consistent LLD group (median = 2.47), W = 357, p = 0.038, r = 0.255 (see Fig. 2e).

## 3 Study 2: Concordance of Reduced Language Asymmetry across Measurement Instruments

### 3.1 Methods

#### 3.1.1 General procedure, recruitment strategy, and inclusion criteria

All participants from Study 1 who were consistently classified as bilateral language dominant (cBLD) based on the verbal fluency task in both Doppler sessions, as well as a group of consistently left language dominant controls (cLLD) completed a visual half-field word reading task to measure language asymmetry behaviorally. The same cohort also participated in an MRI session, repeating the covert letter verbal fluency task during scanning. Additionally, verbal fluency performance was assessed outside the scanner by having participants name as many words as possible beginning with a given letter (k, n, or a) within 1 min. The MRI session was scheduled at least 5 days after the second Doppler session.

#### 3.1.2 Visual half-field task procedure and data processing

A visual half-field task (VHT), adapted from Van Der Haegen et al. (2011), was used to obtain a behavioral measure of hemispheric asymmetry. In this task, a target word displayed in either the left or the right visual hemifield had to be repeated out loud. Because the primary visual cortex receives input from the contralateral visual field, the language-dominant hemisphere will have preferential access to words presented to the opposite hemifield, resulting in faster and more accurate processing of verbal information in the visual field opposite to the dominant hemisphere. The task was programmed and presented using PsychoPy version 2021.2.3.

The verbal stimulus material included 216 Dutch common nouns, each consisting of three or four letters. The outer edge of the words was offset by 3.39° and their inner set by 2.07° (three letters) or 1.6° (four letters) with respect to the middle of the screen. Half of the words acted as targets, while the other half were presented on the opposite side of the target to serve as fillers. This bilateral mode of stimulus presentation ruled out lateralized attentional effects resulting from the abrupt appearance of a single item in one half field only (Bourne, 2006). Each trial started with a central fixation cross shown for 500 ms, after which a word pair appeared and the fixation cross was replaced by an arrow pointing at the target word. The two words and arrow remained on the screen for 200 ms to minimize saccades that would interfere with the lateralized mode of presentation (Bourne, 2006). Next, a series of hashtags was shown for 200 ms to eliminate afterimages that could have prolonged the presentation duration of the words, after which the fixation cross reappeared for 1.200 ms before the next trial started. Participants were asked to overtly read the target word as fast as possible, yet accurately. Twenty-four fixation control trials were interspersed between the experimental trials to motivate participants to maintain central fixation. These control trials consisted of a single digit symbol shown for 80 ms in the center of the screen which participants had to say out loud as quickly as they could. Stimuli were organized in blocks of 60 trials and each block was separated by a self-paced break. Target-filler pairs were counterbalanced across presentation side such that each target appeared once in the left and once in the right hemifield. At the start of the experiment participants completed a practice block consisting of 16 trials. All verbal responses were recorded using the build-in audio capture feature of PsychoPy.

#### 3.1.3 fMRI data collection and processing

MRI data were collected on a 3.0-T Prisma scanner (Siemens) with a 64-channel head coil. First, a T1 MPRAGE image with whole-brain coverage was acquired with the following parameters: 1 mm isotropic voxel size, 176 sagittal slices, repetition time [TR] = 2.250 ms, echo time [TE] = 4.18 ms inversion time [TI] = 900 ms, flip angle = 9°. A fieldmap for EPI distortion correction was acquired prior to functional imaging using a double-echo gradient echo field map sequence consisting of two magnitude images and a single phase difference image. Functional BOLD imaging was done using a T2-weighted echo planar sequence with parameters: 2.5 mm isotropic voxel size, 60 transversal slice, TR = 1.070 ms, TE = 31 ms, TI = 17 ms, flip angle = 52°, multiband factor = 4.

During the BOLD imaging, participants performed a block-design letter verbal fluency task similar—but not identical—to the one used in the previous Doppler sessions. The fMRI verbal fluency task consisted of two conditions. During the word generation condition, a letter was displayed on the screen and participants had to silently think of words starting with that letter. During the active control condition, participants covertly repeated the pseudoword ‘baba’, which was shown on the computer screen. Each word generation and control trial lasted 15 s and were separated by a 15 s resting period. Seven trials per condition were presented.

The MRI data were preprocessed using *fMRIPrep* 23.2.1 (Esteban et al., 2018, 2019; RRID:SCR_016216), which is based on *Nipype* 1.8.6 (K. Gorgolewski et al., 2011; K. J. Gorgolewski et al., 2018; RRID:SCR_002502). The T1w image was corrected for intensity non-uniformity (INU) with N4BiasFieldCorrection (Tustison et al., 2010), distributed with ANTs 2.5.0 (Avants et al., 2008; RRID:SCR_004757), and used as T1w-reference throughout the workflow. The T1w-reference was then skull-stripped with a Nipype implementation of the antsBrainExtraction.sh workflow (from ANTs), using OASIS30ANTs as target template. Brain tissue segmentation of cerebrospinal fluid (CSF), white-matter (WM), and gray-matter (GM) was performed on the brain-extracted T1w using FAST (FSL (version unknown), RRID:SCR_002823, Zhang et al., 2001). Volume-based spatial normalization to two standard spaces (MNI152NLin2009cAsym, MNI152NLin2009cSym) was performed through nonlinear registration with antsRegistration (ANTs 2.5.0), using brain-extracted versions of both T1w reference and the T1w template. The following template was selected for spatial normalization and accessed with TemplateFlow (23.1.0, Ciric et al., 2022): ICBM 152 Nonlinear Asymmetrical template version 2009c [Fonov et al., 2009, RRID:SCR_008796; TemplateFlow ID: MNI152NLin2009cAsym]. To process the functional data, first, a reference volume was generated, using a custom methodology of fMRIPrep, for use in head motion correction. Head-motion parameters with respect to the BOLD reference (transformation matrices, and six corresponding rotation and translation parameters) were estimated before any spatiotemporal filtering using mcflirt (FSL, Jenkinson et al., 2002). A B0 nonuniformity map (or fieldmap) was estimated from the phase-drift map(s) measure with two consecutive GRE (gradient-recalled echo) acquisitions. The corresponding phase-map(s) were phase-unwrapped with prelude (FSL None). The estimated fieldmap was then aligned with rigid-registration to the target EPI (echo-planar imaging) reference run. The field coefficients were mapped onto the reference EPI using the transform. The BOLD reference was then co-registered to the T1w reference using mri_coreg (FreeSurfer) followed by flirt (FSL, Jenkinson & Smith, 2001) with the boundary-based registration (Greve & Fischl, 2009) cost-function. Co-registration was configured with six degrees of freedom.

The normalized BOLD series were further processed into SPM12 implemented in Matlab version 2019b. Gaussian smoothing was performed using a 5 mm kernel. Next, for each participant, a general linear model was estimated that included the task onsets convolved with the canonical hemodynamic response function alongside the six motion realignment parameters as nuisance regressors.

Participant-specific LIs were calculated within a region-of-interest (ROI) covering the MCA territory (see Fig. 4a) using the threshold-independent bootstrapping procedure implemented in the LI-toolbox (Wilke & Lidzba, 2007; Wilke & Schmithorst, 2006). The resulting LIs range from -1 (only activation in the left hemisphere) to +1 (only activation in the right hemisphere), with a score of zero indicating perfectly symmetrical activation within the MCA ROI. LIs were calculated for two contrasts: verbal fluency > BABA (active control) and verbal fluency > rest (passive control). Using an active baseline is the default approach in fMRI, whereas the contrast with a passive baseline better matches with the way fTCDS data are conventionally analyzed.

#### 3.1.4 Statistical analysis

The concordance between the fTCDS LIs (see Study 1) and asymmetry measures obtained from the visual half-field task and fMRI was assessed using Spearman’s rank correlations. To examine whether consistent bilaterality based on fTCDS generalizes to other methods, we compared the absolute value of the visual half-field asymmetry and fMRI LI between cBLD and cLLD participants using one-sided Man-Whitney U tests, hypothesizing a lower degree of asymmetry in the cBLD group. Non-parametric statistics were chosen because the distribution of LIs tends to be highly skewed.

### 3.2 Results

#### 3.2.1 Participant sample

All participants consistently classified as BLD in the two Doppler sessions (cBLD, n=18) as well as 19 consistently classified left language dominant controls (cLLD) completed a visual half-field task and the fMRI version of the letter verbal fluency task. The participant characteristics are shown in Table 2. The cBLD and cLLD participants did not differ in terms of age, sex, handedness, and number of words produced during the out-of-scanner letter verbal fluency task. Due to logistical reasons, the time between the second Doppler session and the scanning session was significantly longer for the participants in the cBLD group (mean = 68.5 days, range = 6 to 203 days) compared to participants in the cLLD group (mean = 26.6, range = 5 to 197 days).

**Table 2.** Demographics of participants in Study 2.

	cBLD	cLLD	Group comparison	
	(n = 18)	(n = 19)	Statistic	p-value
Age, mean ± sd [years]	21 (4.2)	20.1 (2.8)	t(29.4) = 0.757	0.455
Sex [% Female]	88.9%	78.9%	Χ²(1) = 0.140	0.709
Handedness [% Left]	72.2%	73.7%	Χ²(1) < 0.001	1.000
Verbal fluency score [N words]	39.5 (14.2)	38.9 (9.5)	t(29.5) = 0.138	0.891
Days between MRI and 2d fTCDS	68.5 (65.5)	26.6 (43.2)	**t(29.2)** **=** **2.82**	**0.030**

cBLD and cLLD = participants consistently classified as bilateral and left language dominant respectively based on the two Doppler sessions. Values in bold indicate statistical significance (p < 0.05).

#### 3.2.2 Visual half-field task

**Fig. 3.**
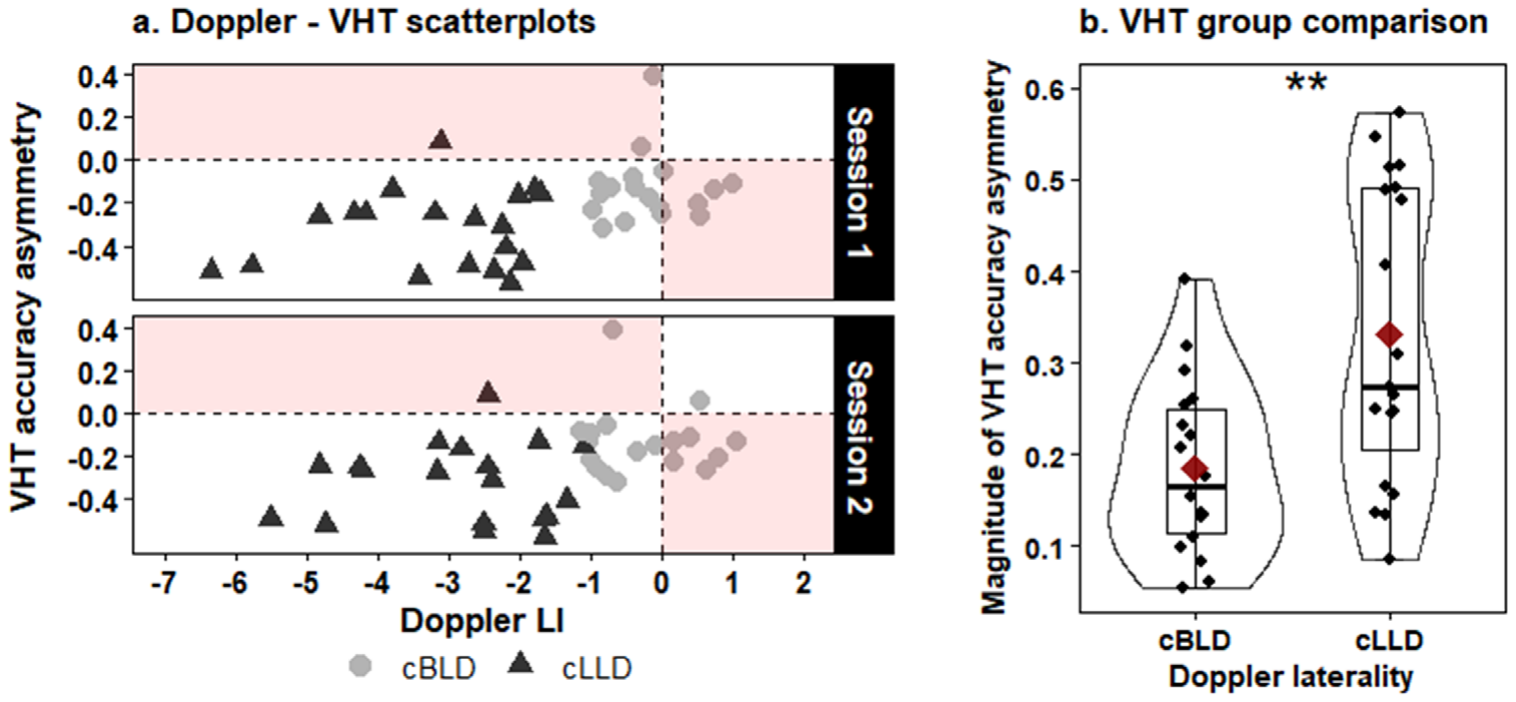
Correspondence between Doppler and visual half field measures of language asymmetry. (a) Scatterplot of the LIs obtained during the Doppler sessions and asymmetries observed during the visual half-field task (VHT). Negative values indicate leftward asymmetry, positive values indicate rightward asymmetry. Shaded areas represent discrepancies between Doppler and VHT laterality. (b) Comparison of the magnitude (i.e. absolute value) of the VHT accuracy asymmetry between participants consistently classified as bilateral (cBLD) or left lateralized (cLLD) based on the two Doppler test sessions. The boxplots span the 25^th^ to 75^th^ percentile, with the median indicated by the thick black line. The red diamond indicates the mean. **p < 0.01.

Significant positive correlations were found between the visual half-field asymmetry and the LI ‘s obtained from the first Doppler session (ρ = 0.5, p = 0.002) and the second Doppler session (ρ = 0.44, p = 0.007) (see Fig. 3a). The absolute value of the visual half-field asymmetry was significantly lower in the cBLD group (median = 0.166) compared to the cLLD group (median 0.272) according to a one-sided Mann Whitney U test, W = 78, p = 0.002, r = 0.464 (see Fig. 3b).

#### 3.2.3 fMRI

**Fig. 4.**
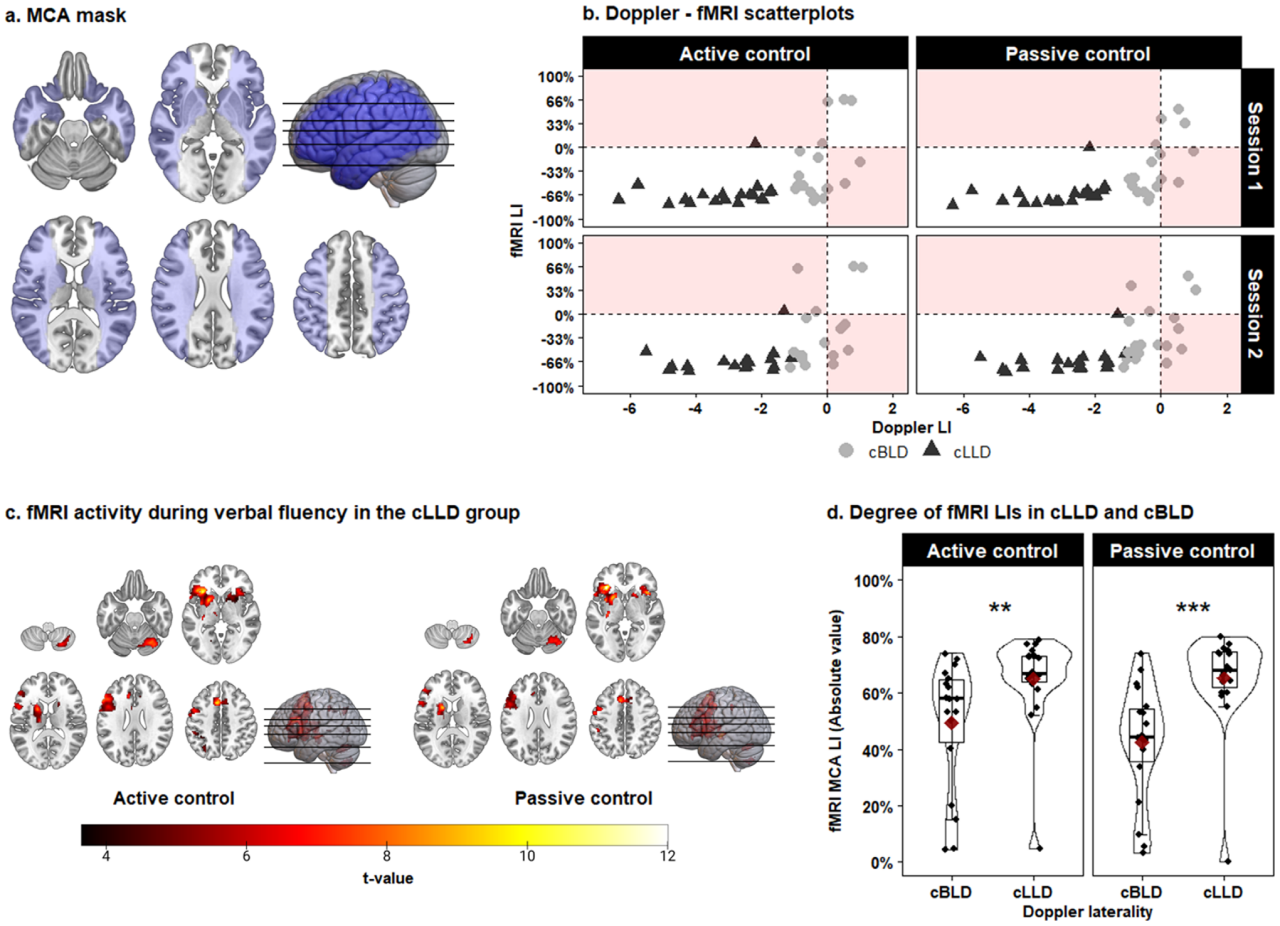
Correspondence between Doppler-based and fMRI-based laterality indices within the MCA vascular territory. (a) Cortical regions irrigated by the middle cerebral artery (CBA). (b) Scatterplots of LIs derived from the Doppler and fMRI verbal fluency tasks. fMRI LIs are calculated within the MCA mask and are shown separately for participants consistently classified as bilateral (cBLD) and left lateralized (cLLD) across the two Doppler test sessions. Shaded areas represent discrepancies between Doppler and fMRI laterality. (c) Significant group fMRI activation in the cLLD group, P cluster FDR < 0.05, P peak uncorrected < 0.001. (d) Comparison of the magnitude of the fMRI LIs between cLLD and cBLD groups. The boxplots span the 25^th^ to 75^th^ percentile, with the median indicated by the thick black line. The red diamond indicates the mean. **p < 0.01, ***p < 0.001.

Significant positive correlations were observed between the fMRI LIs and the fTCDS LIs from both Doppler sessions, irrespective of whether the fMRI LIs were calculated with an active or passive baseline (see Table 3 and Fig. 4b). Activation maps from the fMRI task contrasting the verbal fluency condition with the active or passive baseline conditions are shown in Figure 4c, and the locations of peak activation are listed in Supplementary Materials 3. The absolute value of the fMRI LIs were significantly lower in the cBLD group (median active baseline = 0.58, median passive baseline = 0.445) compared to the cLLD group (median active baseline = 0.67, median passive baseline = 0.68) based on a one-sided Mann Whitney U test (active baseline: W = 76.5, p = 0.004, r = 0.472; passive baseline: W = 46.5, p < 0.001, r = 0.623) (see Fig. 4d).

**Table 3.** Spearman’s Rho correlations between the LIs of the Doppler and fMRI sessions.

fTCDS session	fMRI baseline	ρ	P-value
Session 1	Active	**0.63**	**<0.001**
	Passive	**0.66**	**<0.001**
Session 2	Active	**0.75**	**<0.001**
	Passive	**0.75**	**<0.001**

Values in bold indicate statistical significance (p < 0.05).

## 4 Discussion

This study investigated the test-retest reliability of small LIs in CBFV during letter verbal fluency (Study 1) and examined if consistent bilateral classification based on these LIs indicates that the reduced asymmetry generalizes across other cerebral laterality measures (Study 2). Many prior validation studies have reported solid test-retest reproducibility of task-evoked hemodynamic techniques for assessing language laterality, particularly for expressive language tasks (Bishop et al., 2009; Bradshaw et al., 2017; Jansen et al., 2006; Nettekoven et al., 2018; Rutten et al., 2002; Stroobant & Vingerhoets, 2001; Wilson et al., 2017). In line with those studies, we observed favorable test-retest reliability in our subgroup of participants initially classified as left language dominant, who showed a moderately high intraclass correlation score and a near-perfect agreement in their classification between the two test sessions. Only a single participant switched from strongly left dominant in the first session to (left) bilateral in the follow-up session. Enriching our dataset with uncommon individuals with small LIs, who are present to a limited extent or entirely absent in samples of previous studies, allowed us to expand the current literature by confirming previous suspicions that weak asymmetries tend to replicate poorly (Carey & Karlsson, 2019; Vingerhoets et al., 2023). Equally, classifying participants as bilateral turned out to be not particularly reproducible across sessions, regardless of whether a data-driven or cutoff-based categorization scheme was used. It is, nevertheless, notable that initial bilateral participants whose classification did not reproduce still displayed, on average, a significantly lower degree of cerebral laterality during the retest Doppler session compared to consistently left lateralized controls. Follow-up analyses enabled us to rule out that poorer reproducibility in participants with small LIs compared to clearly left lateralized LIs could be attributed to differences in fTCDS recording settings, trial-to-trial signal variability, or general retest effects. Participants who were repeatedly classified as bilateral in both fTCDS sessions were significantly less asymmetrical in a visual half-field reading task and a letter verbal fluency fMRI task compared to consistent left language dominant controls. It is important to note, however, that the individual variability in the magnitude of these asymmetries was considerable in both participant groups.

### 4.1 The LI aggregates trait, state and noise components

There is an ongoing debate on whether individual differences in the degree of LIs reflect variations in cerebral asymmetry as a person-specific trait or as state-dependent fluctuations (Hausmann, 2019). Evidence that these LIs are at least partially state-driven comes from demonstrations that situational psychological and physiological processes such as mood, stress, and hormonal oscillations can dynamically influence the strength and sometimes even direction of functional asymmetries (Berretz et al., 2022; Compton & Levine, 1997; Hausmann, 2002, 2017; Hodgetts et al., 2015). Adding to the complexity is that the experimental and instrumental noise inherent to our measurement methods guarantees that the *recorded* activation asymmetry will not perfectly match the true laterality of the neural system at the time of measurement (i.e., hemisphere asymmetry as a *construct*). The within-subject discrepancies we observed between the LIs from the first and second Doppler recordings likely originated from between-session variability in situational influences and measurement noise. While intracranial blood flow velocity is influenced by fluctuating physiological factors such as hematocrit levels, blood pressure, and carbon dioxide levels (Purkayastha & Sorond, 2013), these potential sources of measurement error likely did not affect our Doppler-based LIs as their impact is expected to be systematic across both hemispheres and accounted for through normalization and baseline correction during data preprocessing. Variations in probe positioning and the angle of insonation between sessions, although also in principle corrected for through normalization, could, nevertheless, have introduced inconsistencies in data quality and the location within the MCA from which the signal was sampled, potentially impacting the test-retest reliability (Deppe et al., 2004).

However, our data suggest that the (degree of the) LI is not solely influenced by noise and situational factors, but also by the idiosyncratic asymmetry of the participant’s language system, since reduced asymmetry persisted over time and across measurement modalities in our cBLD subgroup. It is highly unlikely that measurement noise would consistently decrease asymmetry in the same group of individuals, given the considerable variation in technical complexity and reliance on different physiological mechanisms in our methods (CBFV for fTCDS, behavior for the VHT, and BOLD response for fMRI). Similarly, differences in situational factors cannot explain the sustained group-level reduction of asymmetry over test sessions in cBLD participants either, as the exact circumstances and situational influences during different test days can never be identical. Therefore, we propose that hemodynamic-based LIs are shaped by the interplay of situational influences, noise components, and the inherent, individual-specific cerebral asymmetry. This conclusion mirrors those of previous studies showing that electroencephalography alpha asymmetry at rest encompasses both state and trait factors (Hagemann, 2004; Hagemann et al., 2005).

Approaching the LI as a mixture of trait, state, and noise components may offer an explanation as to why smaller LIs reproduced more poorly across test sessions compared to larger LIs in our study. Assuming that the absolute asymmetry shifts induced by situational and noise factors are independent of someone’s trait cerebral laterality, the LIs of individuals who are naturally inclined toward more symmetrically balanced activation will be disproportionally impacted compared to people with inherently more lateralized networks. Similarly, because the range of LIs typically classified as bilateral is more limited than those deemed as lateralized, circumstantial fluctuations can more readily push a participant’s recorded asymmetry over classification boundaries when they tend to produce relatively weaker brain asymmetry.

### 4.2 “Bilateral” LIs generally represent “weak” rather than absent laterality

What are the implications of bilateral LIs for the hemispheric organization of language? Some researchers have understood these to signify a complete absence of hemispheric specialization (e.g., Knecht et al., 2000; Mazoyer et al., 2014), akin to the various patterns of bilateral language representation observed in patients during Wada testing (Bernal & Ardila, 2014). The notable similarities in the prevalence of bilaterality and its association with handedness observed through the Wada procedure and neuroimaging studies might suggest that bilateral outcomes in both methods reflect the same type of hemispheric organization for language. For instance, McManus (2022) recently integrated seven large-scale Wada studies (n=173 to 551 participants) to estimate that bilateral language representation occurs in 10.4% of right-handers and 21.8% of non-right-handers (Bauer et al., 2014; Janecek, Swanson, Sabsevitz, Hammeke, Raghavan, Mueller, et al., 2013; Kurthen et al., 1994; Loring et al., 1990; Möddel et al., 2009; Rasmussen & Milner, 1977; Risse et al., 1997), which matches the proportions we observed in the current fTCDS study (10.2% of right-handers and 20.2% of left-handers) and those reported by other big sample functional imaging studies (e.g., Basic et al., 2004; Jansen et al., 2007; Mazoyer et al., 2014). Nevertheless, one must be careful in assuming a one-on-one mapping between bilaterality based on Wada testing and based on neuroimaging. Although fMRI evaluations of language laterality usually align with those derived from the Wada procedure, results are highly discordant when either method indicates bilateral language dominance (Janecek, Swanson, Sabsevitz, Hammeke, Raghavan, Rozman, et al., 2013). Discordances could arise when situational factors impact task-evoked activation asymmetry or the ensuing hemodynamic response, but do not interfere with the performance on the Wada test. One such factor could be the hormonal fluctuations that occur monthly as part of the menstrual cycle (Hausmann, 2002, 2017). A noteworthy single case study demonstrating this possibility reports on a female patient with epilepsy whose LI varied from clearly left to bilateral-right dominant along the menstrual cycle during repeated testing with fTCDS and fMRI, but remained stably right lateralized across successive Wada examinations (Helmstaedter et al., 2015). Another explanation for these discrepancies between the two methods is that the Wada procedure may overestimate the degree of cerebral laterality when linguistic resources in the non-dominant hemisphere, although present, are insufficient to retain minimal performance when the dominant side is sedated, incorrectly suggesting a complete lack of non-dominant hemisphere involvement in language (Janecek, Swanson, Sabsevitz, Hammeke, Raghavan, Rozman, et al., 2013). Finally, as with hemodynamic imaging, the prevalence of bilaterality in Wada testing depends on which tasks are selected to assess language dominance as well as on how bilateral language dominance is defined (Bernal & Ardila, 2014). For example, in the report on the Wada procedure by Risse et al. (1997), 39 patients were considered bilateral when there was unambiguous evidence of language function in at least one task (automatic speech, naming, auditory comprehension, and reading) following each injection. However, only two of those patients displayed true hemispheric equivalence in language ability, while the rest had overall left or, less commonly, right hemispheric language superiority (Risse et al., 1997).

An alternative interpretation is that (most) individuals with bilateral LIs do not lack hemisphere dominance, but rather have a weakly lateralized system in which one hemisphere still consistently assumes predominant control over the task (Bishop et al., 2024). While our experimental design does not permit us to definitively rule out the absence of hemisphere dominance in our bilateral-classified participants, the observation from Study 1 that laterality rarely flipped sides between Doppler test sessions is more in line with the notion that most of them have at least some degree of hemispheric specialization for verbal fluency. Data from the study of Zago et al. (2017) similarly support that language laterality is generally retained in cases of bilateral LIs. In their study, a machine-learning classifier was trained on fMRI activation maps of sentence production to distinguish the dominant from the non-dominant hemisphere, after which the two hemispheres of out-of-sample participants were independently classified as dominant or non-dominant. When this procedure was applied to participants with a bilateral LI, only 27% (10/37) were assigned patterns indicative of true hemispheric equivalence, in which both hemispheres were classified as dominant or both as non-dominant. The remaining 73% of participants had one hemisphere classified as dominant and the other as non-dominant.

### 4.3 Practical considerations for handling small asymmetries

An important motivation for why we assessed the test-retest reliability of small LIs was to deliver evidence-guided practical considerations for how to treat small asymmetries obtained from hemodynamic neuroimaging, which are currently lacking (Vingerhoets et al., 2023). In what follows, we draw several recommendations from our data, which will depend on the context and specific goal of measuring cerebral laterality. We argue that, in general, the range of valid approaches depend on how defensible it is to sacrifice subject-specific precision of the measurement in favor of retaining bigger and more representative participant samples. When avoiding misclassifications is essential, the safest course of action will be to either exclude participants with small LIs or to repeat the measurement, given their stellar test-retest reproducibility. This is especially relevant in presurgical assessments, where erroneous classifications can be costly. Our data support the latest clinical decision-making recommendations to follow up bilateral activation patterns in neurosurgery candidates with a second measurement, using more invasive procedures for determining hemisphere dominance, such as the Wada test (Campbell et al., 2023; Massot-Tarrús & Mirsattari, 2022; Qadri et al., 2021).

In light of the poor reproducibility of bilaterality as a category, we recommend that studies aiming to better understand the significance of the degree of functional hemisphere asymmetry, such as for behavior or brain anatomy, avoid comparisons between lateralized and bilateral subgroups. Instead, LIs should be correlated with the measures of interest. However, we would also generally caution against excluding participants with ‘small’ LIs. Doing so will restrict the generalizability of the results, as data from individuals who are genuinely less lateralized are discarded. Additionally, this approach risks underestimating real associations between cerebral laterality and the measures of interest by narrowing the variability of asymmetries in the data. If classifying the hemisphere dominance of individual participants is necessary, for example, to estimate rates of (a)typical asymmetry, our data suggest that a simple left-right categorization scheme leads to minimal reliability issues, even in participants with smal LIs, and certainly yields more reproducible outcomes than a trichotomous categorization that adds a bilateral category. Alternatively, rather than relying on a single classification, one could opt to explore rates of (a)typical hemisphere dominance across multiple LI thresholds, including zero, to uncover potential interactions between LI direction and degree.

### 4.4 Limitations

Language is a complex function that engages a broad range of processes, which may not necessarily lateralize to the same extent or even the same hemisphere within an individual (Woodhead et al., 2019). However, the current study only assessed the test-retest reliability of bilaterality during letter verbal fluency. This task is among the most well-established for determining hemisphere language dominance and yields pronounced group-level asymmetries (Bradshaw et al., 2017), which benefited this study by allowing for a large contrast in the magnitude of LIs between bilateral and clearly (left) lateralized participants. The decision to include only this task increased the feasibility of this study given its complex test-retest design, but came at the cost of neglecting the possibility to assess the reliability of asymmetries for other core domains of language, such as syntaxis and verbal comprehension.

Another limitation is that the retest samples in Study 1 and Study 2 ended up imbalanced for sex, including mostly biologically female participants, which may have reduced the generalizability of our results. This imbalance is particularly relevant given the small but consistent sex differences in brain lateralization described in the literature (Hirnstein et al., 2019). Specifically evidence suggests that biological woman, on average, tend to have slightly less pronounced hemispheric specialization across various cognitive domains, including verbal functions. As a result, the predominance of female participants in our sample may have introduced a subtle bias in the overall distribution of LIs, potentially limiting its representativeness of the true distribution in the general population. Furthermore, we did not account for the menstrual cycle phase during testing, either in the design or analysis, in spite of known cycle-dependent fluctuations in the degree of functional cerebral asymmetries (Hausmann, 2002; Helmstaedter et al., 2015). Our study may, therefore, have underestimated the replicability of (bi)laterality, which might have been better if each female participant’s series of examinations were all scheduled to coincide at the same phase in the menstrual cycle.

## 5 Conclusion

The current study examined the test-retest reliability and between-methods generalizability of “bilateral” laterality indices during word generation. While less lateralized participants showed poorer reproducibility, they still tended, on average, toward weaker asymmetry upon re-testing and across measurement modalities compared to clearly left-lateralized individuals. This suggests that laterality indices capture not only state-dependent situational and noise factors but also a trait-like component reflecting a function’s inherent degree of asymmetry, which varies continuously across individuals. Reassuringly, sign flips of LIs were rare, even among participants with weak asymmetries. In light of our observations, we recommend in clinical settings to repeat measurements in cases of small LIs or consider more invasive techniques, such as Wada testing, to minimize misclassifications. In research, correlating LIs with other measures of interest may provide more reliable insights than strict categorical comparisons between strongly and weakly lateralized individuals. Finally, a binary left-right classification of LIs is a valid approach and should be favored over schemes that include an additional bilateral category.

## Supplementary Material

Supplementary Material

## Data Availability

Data and experiment and analysis scripts are made publicly accessible on the study’s OSF page: https://osf.io/2jazv/.
