# Supplementary material for: The reliability of bilateral cerebral laterality for word generation: Who is left in the middle?
Source: Imaging Neurosci (Camb). 2025 Aug 7;3:IMAG.a.102. doi: 10.1162/IMAG.a.102 (PMC12336058; doi:10.1162/IMAG.a.102)
Supplement: Supplementary Material [file IMAG.a.102_supp.pdf]

Supplementary materials 1: Doppler settings

	Mean 1	Mean 2	t-value	df	P
**LH depth session 1**					
cLLD – cBLD	50.81	50.67	0.166	25.7	0.869
cLLD – iBLD	50.81	50.88	-0.104	32.6	0.918
cBLD – iBLD	50.67	50.88	-0.226	29.9	0.822
**RH depth session 1**					
cLLD – cBLD	51.09	49.89	1.025	32.1	0.313
cLLD – iBLD	51.09	50.76	0.405	47.0	0.687
cBLD – iBLD	49.89	50.76	-0.804	24.7	0.429
**LH depth session 2**					
cLLD – cBLD	51.56	51.22	0.408	28.7	0.687
cLLD – iBLD	51.56	50.59	1.558	39.5	0.127
cBLD – iBLD	51.22	50.59	0.740	28.7	0.465
**RH depth session 2**					
cLLD – cBLD	50.84	52.00	-1.322	45.7	0.193
cLLD – iBLD	50.84	52.18	-1.418	40.3	0.164
cBLD – iBLD	52.00	52.18	-0.194	32.0	0.847
**LH gain session 1**					
cLLD – cBLD	41.56	43.33	-1.530	32.6	0.136
cLLD – iBLD	41.56	42.18	-0.405	23.5	0.689
cBLD – iBLD	43.33	42.18	0.693	29.0	0.494
**RH gain session 1**					
cLLD – cBLD	41.91	41.00	0.763	41.6	0.450
cLLD – iBLD	41.91	41.53	0.267	31.4	0.791
cBLD – iBLD	41.00	41.53	-0.365	30.2	0.718
**LH gain session 2**					
cLLD – cBLD	41.84	43.00	-0.913	27.3	0.369
cLLD – iBLD	41.84	42.94	-0.829	24.7	0.415
cBLD – iBLD	43.00	42.94	0.036	32.7	0.971
**RH gain session 2**					
cLLD – cBLD	41.94	41.67	0.224	33.4	0.823
cLLD – iBLD	41.94	41.88	0.044	30.7	0.965
cBLD – iBLD	41.67	41.88	-0.152	32.9	0.880

cBLD and cLLD = participants consistently classified as bilateral and left language dominant respectively based on the the two Doppler sessions. iBLD = participants classified as bilateral in the first Doppler session, but lateralized in the second Doppler session.

Supplementary materials 2: Standard errors

	Mean 1	Mean 2	t-value	df	P
**fTCDS session 1**					
cLLD – cBLD	0.593	0.511	-1.553	38.8	0.129
cLLD – iBLD	0.593	0.617	0.462	39.2	0.647
cBLD – iBLD	0.617	0.511	1.907	32.9	0.065
**fTCDS session 2**					
cLLD – cBLD	0.607	0.591	-0.300	29.5	0.766
cLLD – iBLD	0.607	0.529	-1.667	31.7	0.105
cBLD – iBLD	0.591	0.529	-1.060	32.5	0.297

cBLD and cLLD = participants consistently classified as bilateral and left language dominant respectively based on the the two Doppler sessions. iBLD = participants classified as bilateral in the first Doppler session, but lateralized in the second Doppler session.

Supplementary materials 3: Activation tables

Baseline	MNI Coor. *x,y,z (mm)*	Anatomical label	Size *n voxels*	Peak T	Cluster P *FDR-cor*	Peak P *Uncorr.*
**Active**	-06, 06, 57	L Supplementary motor cortex	1174	12.11	< .001	< .001
	-31, 23, -01	L insula, L pars opercularis and triangularis, L frontal operculum, L putamen	4834	11.89	< .001	< .001
	32, -62, -26	R cerebellum, lobule VI	571	8.42	< .001	< .001
	22, -70, -51	R cerebellum, lobule VIII	353	7.30	< .001	< .001
	-04, 03, 27	L and R cingulate gyrus	50	6.63	.003	< .001
	-29, -65, 37	L Superior parietal lobule, angular gyrus, Supramarginal gyrus	321	5.93	< .001	< .001
	-16, -70, 05	L lingual gyrus	122	4.67	< .001	< .001
	-56, -37, 07	L superior and middle temporal gyrus	52	4.44	.003	< .001
	-51, -57, -11	L inferior temporal gyrus	34	4.02	.013	< .001
**Passive**	-34,26, -01	L insula, L pars opercularis and triangularis, L frontal operculum, L putamen	3363	13.41	< .001	< .001
	-06,08, 65	L Supplementary motor cortex	1054	10.06	< .001	< .001
	41,18, -01	R insula, R pars opercularis and triangularis, R frontal operculum, R putamen	552	9.166	< .001	< .001
	32,-67, -23	R cerebellum, lobule VI	421	7.768	< .001	< .001
	22,-70, -51	R cerebellum, lobule VIII	230	7.114	< .001	< .001
	-49,-35, 45	L Supramarginal gyrus	69	6.426	.004	< .001
	-29,-62, 37	L Superior parietal lobule, angular gyrus	42	4.74	.023	< .001

P cluster FDR < 0.05, P peak uncorrected < 0.001. L = Left hemisphere, R = right hemisphere.
